# Effectiveness of educational intervention based on psychological factors on achieving health outcomes in patients with type 2 diabetes

**DOI:** 10.1186/s13098-018-0368-8

**Published:** 2018-09-03

**Authors:** Arash Salahshouri, Fereshteh Zamani Alavijeh, Behzad Mahaki, Firoozeh Mostafavi

**Affiliations:** 1 Student Research Committee, School of Health, Isfahan University of Medical Sciences, Isfahan, Iran; 2 Department of Health Education and Promotion, School of Health, Isfahan University of Medical Sciences, Isfahan, 8174673461 Iran; 3 Department of Biostatistics, School of Health, Kermanshah University of Medical Sciences, Kermanshah, Iran; 4 Department of Biostatistics, School of Health, Isfahan University of Medical Sciences, Isfahan, Iran

**Keywords:** Psychology, Healthy diet, HbA1c, FBS, Patients, Diabetes mellitus, Type 2

## Abstract

**Background:**

Managing type 2 diabetes (T2D) is assumed to be heavily dependent on patients’ active participation in their own self-care behaviors including prescribed diets.

**Objectives:**

The purpose of the present study was to investigate the effectiveness of educational intervention based on psychological factors on nutritional behaviors as well as levels of fasting blood sugar (FBS) and glycated hemoglobin (HbA1c) in patients with T2D referring to diabetes clinics and healthcare centers in the city of Izeh, Iran.

**Methods:**

A total number of 145 patients were recruited in this clinical trial and then randomly assigned to two groups of intervention (n = 73 individuals) and control (n = 72 individuals). After that, a researcher-made multi-part questionnaire including a demographic characteristics information form, a nutritional perceptions and beliefs questionnaire; a scale measuring fears, concerns, and discomforts associated with diabetic diet, as well as the valid and reliable Perceived Dietary Adherence Questionnaire were used to collect the required data before and 3 months after the completion of the educational intervention. To this end, the patients in the intervention group attended an educational program for eight sessions but the individuals in the control group only received routine services. Data analysis was also conducted using the SPSS Statistics (Version 18) and via descriptive and inferential statistics.

**Results:**

The findings revealed that the mean scores of the sub-groups of nutritional perceptions and beliefs (but not exaggerated ones) in the patients assigned to the intervention group were significantly higher than those in the control group after 3 months (p = 0.001). As well, the mean scores of the sub-groups of fears, concerns, and discomforts in patients as well as exaggerated beliefs witnessed a significant decrease in the intervention group compared to those in the control group (p = 0.001) 3 months after the educational intervention. Furthermore, the mean scores of adherence to a healthy diet in the intervention group had significantly increased compared to those in the control group. There was correspondingly a significant descending trend in the average levels of fasting blood sugar (FBS) and glycated hemoglobin (HbA1c) in the intervention group compared to those obtained in the control group (p = 0.001).

**Conclusion:**

The results of this study shed light on the importance of the effectiveness of psychological factors on achieving health outcomes in patients with type 2 diabetes (T2D). Moreover, a new combination of diet-related psychological factors in patients with diabetes was introduced in the present study.

*Trial registration* IRCT. IRCT20180308039008N1. Registered 15 April 2018, http://www.irct.ir

## Background

According to a report released by the World Health Organization (WHO), 422 million adults in the world were affected with diabetes in 2014 [1], mostly type 2 diabetes (T2D) [1].

According to the International Diabetes Federation’s Diabetes Atlas, 415 million people worldwide, or one in 11 adults, were also suffering from diabetes in 2015 [2], and this figure is expected to reach 592 million by 2035 [3] wherein most patients will have T2D [1, 2]. Although the incidence of T2D varies according to the geographical area, over 80% of these patients are living in low- and middle-income countries [4]. However, the prevalence rate of T2D is estimated by 8.6% in Iran [5].

The management of T2D can be heavily dependent on patients’ active participation in their own self-care behaviors including prescribed diets [6–9]. In this respect, adherence to a healthy diet has been recognized as the first step [10, 11] and the most complicated dimension of diabetes management [12] which can consequently lead to stable improvement in the levels of glycated hemoglobin (HbA1c) [13] and control of blood sugar in patients [14, 15]. In spite of much progress within diabetes management, non-adherence to prescribed treatments including dietary recommendations, is taken into account as a major barrier to the control of blood sugar levels in various countries [16–18] which can even expose patients to risks of seriously chronic complications and early death [18–20]. According to the related literature, low adherence to prescribed diets has been constantly reported in patients with T2D [12, 21, 22]. Studies have similarly concluded that patients’ adherence to a healthy diet can be influenced by numerous factors including psychological ones [23, 24]. One of the cited factors in this respect has been associated with patients’ nutritional perceptions and beliefs playing an important role in determining and changing behaviors in patients with diabetes [25]. Therefore, the results of the relevant investigations have suggested a relationship between attitudes towards diabetes and dietary adherence [26]. Although such studies have less examined fears, concerns, and discomforts associated with dietary adherence in diabetic patients, there is the possibility that the given factors have an effect on patients’ adherence. So, this study was conducted to determine the effectiveness of educational intervention on (a) psychological factors related to dietary adherence and (b) nutritional behaviors as well as levels of fasting blood sugar (FBS) and HbA1c in patients with T2D residing in the city of Izeh, Iran.

## Methods

### Study design

The present study was fulfilled as a randomized controlled clinical trial from November to April 2017. Using a pretest–posttest design, this investigation was to determine the effectiveness of educational intervention based on psychological factors on adherence to a healthy diet as well as levels of FBS and HbA1c in patients with T2D.

### Sampling

**Fig. 1:**
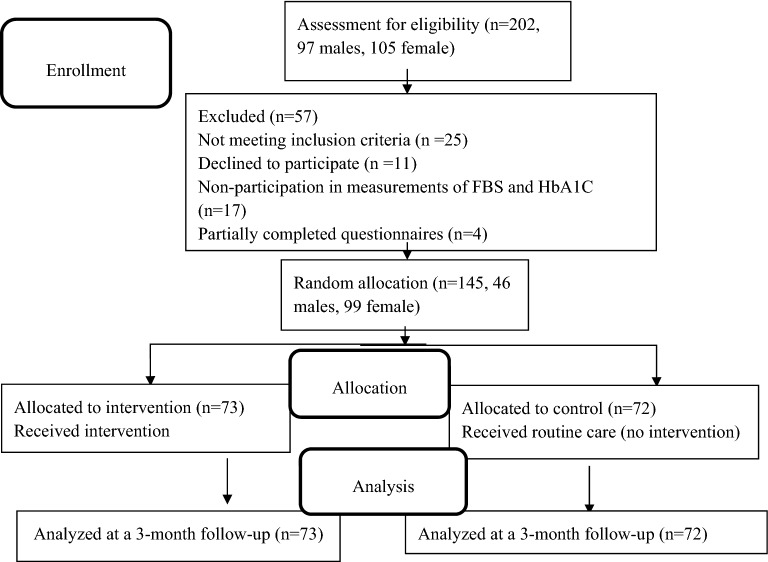
Flow diagram of the participants

The study population was comprised of patients with T2D referring to diabetes clinics and healthcare centers in the city of Izeh. Using the $\frac{{2\left( {z_{1} + z_{2} } \right)^{2} \sigma^{2} }}{{d^{2} }}$ formula as well as taking 0.95 confidence levels, 0.80 test power, and 10% sample loss into account; the final sample size for each group was calculated by at least 70 individuals and a total number of 140 patients. Since most of the patients withdrew from the study after it was started, a total number of 202 patients were included but 57 people out of them were excluded for various reasons (lack of meeting the inclusion criteria, unwillingness to cooperate, refusal to do the tests, and returns of incomplete questionnaires). Thus, 145 patients were ultimately recruited in the study. Sampling was also preformed systematically via the records of diabetic patients. These individuals were also randomly divided into two groups: intervention (73 individuals) and control (72 individuals) based on random number table and selection through drawing (firstly, the list of the individuals’ names was provided; after that, each person was assigned to one of the groups via drawing) (Fig. 1).

The inclusion criteria in this study were the diagnosis of the disease at least 3 months prior to the onset of the study, informed consent to participate in the research, lack of other chronic diseases such as cancer and so on, having active records in the diabetes clinics or healthcare centers, as well as no participation in similar classes offered by healthcare centers. The exclusion criteria also included returns of incomplete questionnaires or refusal to complete them, no participation in educational sessions (less than 50% of sessions), and reluctance to have their blood sugar and HbA1c levels tested.

Although a heterogeneous group (different ethnic groups, age, gender, marital status, levels of education, and socio-economic status) of patients with T2D was to be studied, most patients with T2D were elderly people in the region under study. Given that most of older adults are illiterate in Iran, more illiterate patients were chosen as samples. However, in order to enhance their training, tangible educational methods (such as lectures, questions and answers, patient’s successful experiences, Plate Method, role-plays) were used that did not require a high level of literacy.

### Research tools

The data were collected through a four-part questionnaire and via interviews.

1. The first part included a form containing 13 items related to demographic characteristics information such as age, gender, family history of disease, weight, and the like.
2. The second part of the questionnaire was a researcher-made one evaluating nutritional perceptions and beliefs developed based on a qualitative study with 40 items and 7 dimensions including expected outcomes (12 items), beliefs in the effect of some food stuff on decreasing blood sugar (3 items), beliefs related to the consumption rate of some food stuff (2 items), beliefs about healthy food stuff (4 items), exaggerated beliefs (8 items), spiritual beliefs (4 items), as well as self-efficacy (7 items). The respondents could also show their agreement with the items using a 4-point Likert-type scale (totally disagree 1, disagree 2, agree 3, totally agree 4). Moreover, the quantitative face validity and content validity of the initial questionnaire were measured through asking opinions of 11 experts in different fields. The content validity index (CVI) and the content validity ratio (CVR) of the questionnaire were correspondingly reported by 0.91 and 0.79; respectively. In addition, test–retest method with a 3-week interval was used on 30 patients with diabetes to determine the reliability of the research tool; thereby the reliability coefficient of the questionnaire was obtained by 0.94.
3. The third part of the questionnaire was related to a researcher-made one evaluating fears, concerns, and discomforts about nutritional diet in diabetic patients designed based on a qualitative study. This questionnaire was comprised of 11 items and 4 dimensions including concerns caused by economic difficulties (2 items), fears and concerns associated with complications of adherence to diabetic diet (2 items), concerns resulting from feeling like a burden and inattention by family members (2 items), as well as discomforts brought about by life-changing miseries (2 items). The CVI and the CVR of the questionnaire were also reported by 0.90 and 0.84; respectively. The reliability coefficient of the questionnaire was then calculated by 0.91. As well, scores from 1 to 4 were considered (totally disagree 1, disagree 2, agree 3, totally agree 4) for the questionnaire items based on a 4-point Likert-type scale.
4. The fourth part of the questionnaire was Perceived Dietary Adherence Questionnaire (PDAQ) focused on proper dietary adherence whose validity and reliability had been examined by Asaad et al. [27] in the Canadian population in 2015. The given questionnaire included 9 items based on the Canadian Diabetes Association’s Clinical Nutrition Guidelines designed and extracted from Canada’s Food Guide. This questionnaire was translated into Persian by two individuals good at English and then it was retranslated into English by a third person. After adapting it with the original questionnaire and fixing the problems, the final version was translated once again into English. The items of the PDAQ were based on an 8-point Likert-type scale from 0 to 8. The CVI and the CVR of the questionnaire were also reported by 0.78 and 0.88; respectively. The reliability coefficient of the questionnaire was obtained by 0.74 via test–retest method and with the participation of 30 diabetic patients.

### Research procedure

Random assignment of the patients was done using random number table. It should be noted that a statistics consultant who was not involved in the recruitment of participants conducted the given randomization.

The questionnaires were completed by the researchers or interviewers through interviews or by the participants themselves. Moreover, measurements related to height and weight were performed and recorded in the questionnaire. After that, FBS and HbA1c tests were conducted for patients free of charge by a laboratory and the test values were recorded in the questionnaire. After collecting and analyzing the pretest data, the educational intervention program was designed and implemented. Following the fulfillment of the intervention, the researchers tried to be in contact with the participants by telephone for 2 months to respond to their questions and to fix their problems. Three months after the intervention, the questionnaires were recompleted by both control and intervention groups and then the measurements related to height, weight, FBS and HbA1c levels were carried out. In order to prevent patients’ withdrawal from the study, the tests were performed free of charge.

### Interventions

The educational sessions were held in the meeting hall of the healthcare center in the city of Izeh. It should be noted that the educational intervention was designed and implemented based on psychological factors (perceptions, beliefs, fears, concerns, and discomforts towards diabetic diet) because the given factors had been identified as important determinants of nutritional behaviors in diabetic patients in the initial and qualitative phases of this study. The educational intervention was also administered by a team of internal specialists, dietitians, diabetes experts, a psychologist, as well as a religious expert. To this end, the intervention group received the required education within 8 one-hour sessions and in groups of 23–24 individuals.

The content and strategies used in these 8 educational sessions were as follows:

*Session one* the educational sessions started by providing patients with proper information focused on identifying healthy food stuff and amount of energy released after using per gram of carbohydrates, fats, and proteins to improve their beliefs about health as well as the quantitative features of a diabetic diet. Since individuals could learn new behaviors based on the observation of their results as well as the repetition of the ones by a third person or a role model, the strategy of social comparison and outcome feedback was also used to enhance the expected outcomes in this study. *Sessions two and three* to increase self-efficacy, the researchers focused on patients’ successful experiences (vicarious experiences), verbal persuasion by patients’ families and other patients, improvement of skills contributing to patients’ adherence to a healthy diet (Plate Method), as well as skills of saying “no”, leaving tempting situations in parties, and offers of unhealthy foods by those around. Since depression could be considered as one of the barriers to the ability of patients to demonstrate their behaviors; communication skills training, heart-to-heart chit-chats, reduction of negative emotions, correction of mental errors, as well as replacement of positive thoughts were also used to improve it. *Session four* training for the replacement of irrational thinking with logical one (such as teaching not to use emphatic words like “never” in choosing food stuff) was similarly employed to adjust the exaggerated beliefs. *Session five* teaching religious laws on the necessity of attention to health and self-care activities was also performed in cooperation with religious experts to moderate patients’ spiritual beliefs. *Session six* the patients received counseling in the domains of family economic management to address concerns about their problems in this domain. Furthermore, skills training in terms of preparing healthy and low-cost foods were underscored with the help of a dietitian. *Session seven* successful experiences of the patients in controlling the complications of the disease (vicarious experiences) were also used to reduce fears and concerns regarding the complications of a diabetic diet. In order to cope with the concerns caused by life-changing miseries, patients became familiar with the philosophy of life and death in this world and received training focused on coping and performance skills against grief and unhappiness. *Session eight* feedback of family support from patients was also employed to lessen the concerns associated with feeling like a burden and inattention by family members (through playing videos taken from interviews with family members by the research team). It should be noted that these educational sessions were held in the form of lectures, questions and answers, role-plays, educational video playbacks, as well as verbal encouragements and persuasions.

### Statistical methods

The data analysis was performed using the SPSS Statistics (Version 18). Since the collected data were not normally distributed, nonparametric tests were used to analyze and compare the variables. In order to compare the given variables in each group, Wilcoxon signed-rank test was employed. As well, Mann–Whitney U test and Spearman’s rank-order correlation were used to compare these variables between the two groups before and after the educational intervention and to examine the correlation between the variables; respectively.

### Ethical considerations

The Ethics Committee of Isfahan University of Medical Sciences approved the present study (Code of Ethics: IR.MUI.REC.1396.3.522). The consent of the relevant authorities was also obtained before beginning the study. Then; methodology as well as the advantages and disadvantages of the study were explained to them. The participants were assured that they were not obliged to participate in the research and they could withdraw whenever they wished. They were also assured of the confidentiality of their information.

### Findings

The mean age of the patients in the intervention group was by 55.93 ± 12.04 years and that was 54.53 ± 9.43 years in the control group. No significant difference was also observed between both study groups for all the demographic variables (Table 1).

**Table 1:** Demographic characteristics of the study participants in both intervention and control groups before the intervention

Variable	Group	Intervention group mean (standard deviation) frequency (percentage)	Control group mean (standard deviation) frequency (percentage)	p-value
Age		55.93 (12.4)	54.53 (9.43)	0.436
Gender	Male	24 (32.9)	22 (30.6)	0.764
	Female	49 (67.01)	50 (69.4)	
Disease duration		6.05 (4.46)	6 (6.99)	0.241
Family history of disease	Yes	40 (54.8)	39 (54.2)	0.939
	No	33 (45.2)	33 (45.8)	
Marital status	Single, divorced, deceased spouse	4 (5.5)	1 (1.4)	0.366
	Married	69 (54.5)	71 (98.6)	
BMI		27.65 (3.08)	27.49 (3.79)	0.778
Employment status	Employed	19 (26.02)	18 (25)	0.820
	Stay-at-home	46 (63.01)	48 (66.66)	
	Unemployed	8 (10.95)	6 (8.33)	
Level of income	Less than 1 million tomans (250 US dollars)	26 (35.6)	27 (37.5)	0.601
	1 to 1.5 million tomans (250–375 US dollars)	30 (41.1)	33 (45.8)	
	More than 1.5 million tomans (375 US dollars)	17 (23.3)	12 (16.7)	
Place of residence	City	47 (64.4)	47 (65.3)	0.910
	Village	26 (35.6)	25 (34.7)	
Level of education	Illiterate	39 (53.4)	43 (59.7)	0.365
	Lower than high school diploma	20 (27.4)	24 (33.3)	
	High school diploma and higher	14 (19.2)	5 (7)	
Life network	Alone	3 (4.1)	2 (2.8)	0.660
	Living with family	70 (95.9)	70 (97.2)	

The mean scores of perceptions and beliefs related to healthy nutritional behaviors in the studied groups before and after the intervention were illustrated in Table 2. Based on the results, the mean scores of “beliefs in the effect of some food stuff on the neutralization of sugar intake of other ones” before the educational intervention showed a significant difference between the intervention and control groups (54.11 and 49.31, respectively; p-value = 0.045). However, no significant difference was observed between both groups in terms of other variables (p-value > 0.05). According to the given results, all of these variables were reported significantly different between the two study groups after 3 months following the intervention (p-value < 0.001).

**Table 2:** Results of the effectiveness of educational intervention on nutritional beliefs and perceptions in T2D patients

Variable	Time group	Baseline mean ± SD	3-month follow-up mean ± SD	p-value*
Expected outcomes	Intervention	51.51 ± 7.87	82.91 ± 9.02	0.000
	Control	49.51 ± 7.74	49.80 ± 5.24	0.775
	p-value**	0.215	0.000	–
Beliefs in the effectiveness of some food stuff on neutralization of sugar intake of other ones	Intervention	54.11 ± 13.47	78.08 ± 11.66	0.000
	Control	49.31 ± 14.18	51.04 ± 8.61	0.115
	p-value**	0.045	0.000	–
Beliefs related to consumption rate of some food stuff	Intervention	51.20 ± 14.61	81.51 ± 13.84	0.000
	Control	49.48 ± 13.83	49.65 ± 9.38	0.840
	p-value**	0.472	0.000	–
Beliefs related to healthy foods	Intervention	53.36 ± 9.47	84.25 ± 9.12	0.000
	Control	50.97 ± 11.22	52.29 ± 9.19	0.221
	p-value**	0.342	0.000	–
Exaggerated beliefs	Intervention	69.56 ± 11.79	43.96 ± 9.16	0.000
	Control	70.62 ± 11.84	70.40 ± 12.12	0.839
	p-value**	0.655	0.000	–
Spiritual beliefs	Intervention	59.59 ± 14.92	80.91 ± 11.55	0.000
	Control	59.46 ± 16.18	60.76 ± 12.94	0.224
	p-value**	0.890	0.000	–
Self-efficacy	Intervention	47.75 ± 8.76	78.67 ± 10.15	0.000
	Control	48.61 ± 8.47	49.06 ± 7.72	0.658
	p-value**	0.390	0.000	–

* Wilcoxon signed-rank test, ** Mann–Whitney U test

As well, the results of the Wilcoxon signed-rank test revealed that the mean scores of expected outcomes, beliefs in the effect of some food stuff on the neutralization of sugar intake of other ones, beliefs related to the consumption rate of some food stuff, beliefs about healthy foods, spiritual beliefs, and self-efficacy in the intervention group had a significant increasing trend, 3 months after the intervention (p-value < 0.001); but the given values did not change significantly in the control group 3 months after the completion of the educational intervention (p-value > 0.05). Moreover, it was found that the mean scores of exaggerated beliefs in the intervention group after 3 months following the end of the educational intervention had significantly decreased (p-value < 0.001).

The mean scores of fears, concerns, and discomforts associated with adherence to a diabetic diet in the studied groups before and after the intervention were shown in Table 3. Based on the results, no significant difference was reported between the two intervention and control groups before the educational intervention (p-value > 0.05); however, the mean scores of all the mentioned variables indicated a significant difference between both groups after 3 months (p-value < 0.001). Furthermore, the results of the Wilcoxon signed-rank test revealed that the mean scores of all the variables examined 3 months after the completion of the intervention in the intervention group had significantly declined (p-value < 0.001).

**Table 3:** Results of the effectiveness of educational intervention on fears, concerns, and discomforts related to dietary adherence in T2D patients

Variable	Time group	Baseline mean ± SD	3-month follow-up mean ± SD	p-value*
Concerns caused by economic difficulties	Intervention	71.75 ± 20.73	46.06 ± 16.25	0.000
	Control	75.00 ± 16.39	74.13 ± 16.56	0.425
	p-value**	0.455	0.000	–
Fears and concerns about complications of adherence to a diabetic diet	Intervention	71.23 ± 15.65	42.53 ± 9.17	0.000
	Control	72.99 ± 11.09	72.64 ± 11.13	0.965
	p-value**	0.674	0.000	–
Concerns about feeling like a burden and inattention by family members	Intervention	67.47 ± 20.60	42.98 ± 13.82	0.000
	Control	68.40 ± 16.78	67.36 ± 14.47	0.371
	p-value**	0.890	0.000	–
Discomforts caused by life-changing miseries	Intervention	72.77 ± 16.32	47.09 ± 14.06	0.000
	Control	72.57 ± 18.37	69.79 ± 17.27	0.057
	p-value**	0.876	0.000	–

* Wilcoxon signed-rank test, ** Mann–Whitney U test

Based on the results, no significant difference was observed between the mean scores of adherence to a healthy diet as well as levels of FBS and HbA1c in the intervention and control groups before the intervention (p-value > 0.05). However, a significant difference was reported between both groups in terms of the given variables, 3 months after the end of the intervention (p-value < 0.001).

The results also showed that the mean scores of adherence to a healthy diet in the intervention group before the intervention was 37.94 which increased by 54.77, 3 months after the intervention and such a rising trend was statistically significant (p-value < 0.001). However, such a difference in the control group was not reported statistically significant (p-value = 0.059). The mean scores of FBS levels in both intervention and control groups also decreased significantly (3 months after the end of the intervention) (p-value < 0.001). Correspondingly, the results demonstrated that the mean scores of HbA1c in the intervention group had significantly dropped (3  months after the intervention) (p-value < 0.001). Nevertheless, such values increased significantly, 3 months after the end of the intervention in the control group (p-value = 0.024) (Table 4).

**Table 4:** Results of the effectiveness of educational intervention on adherence to a healthy diet as well as changes in levels of FBS and HbA1c in patients in study groups before and after intervention

Variable	Time group	Baseline mean ± SD	3-month follow-up mean ± SD	p-value*
Adherence to a healthy diet	Intervention	37.94 ± 12.85	54.77 ± 8.67	0.000
	Control	36.02 ± 9.73	37.21 ± 9.12	0.059
	p-value**	0.458	0.000	–
FBS	Intervention	147.97 ± 51.56	113 ± 24.08	0.000
	Control	160.57 ± 60.90	147.82 ± 48.79	0.000
	p-value**	0.225	0.000	–
HbA1c	Intervention	7.15 ± 1.68	6.18 ± 0.90	0.000
	Control	7.46 ± 1.89	8.13 ± 8.01	0.024
	p-value**	0.304	0.000	–

* Wilcoxon signed-rank test, ** Mann–Whitney U test

Besides, the results of the Spearman’s rank-order correlation showed a significantly direct relationship between adherence to nutritional behaviors and expected outcomes, beliefs in the effectiveness of some food stuff on the neutralization of sugar intake in other ones, beliefs related to the consumption rate of some food stuff, beliefs about healthy foods, spiritual beliefs, and self-efficacy. The findings further showed a significantly inverse correlation between adherence to a healthy diet and exaggerated beliefs, as well as fears, concerns, and discomforts associated with adherence to a diabetic diet. In addition, the strongest correlation was found between adherence to a healthy diet and exaggerated beliefs (Table 5).

**Table 5:** Correlation matrix of study variables and adherence to a healthy diet before intervention

	1	2	3	4	5	6	7	8	9	10	11	12
1. Expect outcomes	1											
2. Beliefs in the effectiveness of some food stuff on the neutralization of sugar intake in other ones	0.796*	1										
3. Beliefs related to the consumption rate of some food stuff	0.789*	0.804*	1									
4. Beliefs about healthy foods	0.803*	0.786*	0.793*	1								
5. Exaggerated beliefs	− 0.746*	− 0.666*	− 0.673*	− 0.665*	1							
6. Spiritual beliefs	0.667*	0.634*	0.615*	0.620*	− 0.576*	1						
7. Self-efficacy	0.805*	0.727*	0.733*	0.779*	− 0.663*	0.677*	1					
8. Concerns caused by economic difficulties	− 0.605*	− 0.607*	− 0.632*	− 0.700*	0.575*	− 0.488*	− 0.654*	1				
9. Fears and concerns about the complications of adherence to a diabetic diet	− 0.769*	− 0.709*	− 0.708*	− 0.768*	0.722*	− 0.664*	− 0.763*	0.692*	1			
10. Concerns about feeling like a burden and inattention by family members	− 0.622*	− 0.628*	− 0.644*	− 0.608*	0.646*	− 0.571*	− 0.639*	0.569*	0.713*	1		
11. Discomfort caused by life-changing miseries	− 0.552*	− 0.504*	− 0.565*	− 0.522*	0.595*	− 0.545	− 0.614*	0.522*	0.617*	0.659*	1	
12. Adherence to a healthy diet	0.703*	0.604*	0.645*	0.700*	− 0.705*	0.536*	0.699*	− 0.628	− 0.699*	− 0.587*	− 0.515*	1

* Correlation was significant at the 0.01 level (2-tailed)

## Discussion

Understanding psychological factors related to dietary adherence in patients with T2D is likely to contribute to the development of interventions and the improvement of the given factors as a mechanism to change nutritional behaviors in this population. Therefore, the purpose of this study was to determine the effectiveness of educational intervention based on psychological factors on nutritional behaviors as well as the levels of FBS and HbA1C in patients affected with T2D.

In general, the findings of this study showed that the educational intervention had led to positive effects on patients’ perceptions and beliefs 3 months after implementation. Since the higher expectations of individuals about the outcomes of an action could increase the probability of demonstrating the cited behaviors [28] and given that the mean scores of adherence to a healthy diet in the patients had significantly augmented following the educational intervention, it seemed that this intervention had been able to effectively improve their nutritional behaviors through enhancing the expected outcomes. Although the results of the study by Chlebowy et al. [29] did not shed light on a relationship between expected outcomes and self-care behaviors in patients with T2D, the findings of the investigation by Williams and Bond revealed that the expected outcomes had moderated the relationship between self-efficacy and self-care behaviors in a way that the combination of self-efficacy and strong beliefs in the outcomes could prove to be more effective. The results of the present study also demonstrated a significantly positive relationship between patients’ self-efficacy and expected outcomes [30]. In fact, having maximum self-management required positive expectations about outcomes as well as confidence in one’s abilities [31]. As well, the results of the present study illustrated a significantly positive correlation between perceived self-efficacy and adherence to a healthy diet in patients with diabetes. In line with the given results, the study by Reisi et al. [32] and Didarloo et al. [33] showed a significantly positive relationship between self-care behaviors and self-efficiency in patients with T2D. Thus; being consistent with the findings of other investigations [34, 35], the results of the present study could be a documented scientific evidence on the effectiveness of self-efficacy, expected outcomes, and strategies to improve them on increased adherence to a healthy diet. As a result, it could be acknowledged that the intervention had become successful in aiding patients feel that they could achieve positive physical and mental outcomes through dietary adherence. Accordingly, it was recommended to implement interventions based on a simultaneous combination of self-efficacy and expected outcomes.

Numerous studies had similarly underscored the effectiveness of beliefs on self-care behaviors [36, 37]. In this respect, the results of the study by Munni et al. on diabetic mothers showed that 44–58% of them believed that they had to avoid eating some types of fish due to their effects on increasing excessive fetal activity, congenital anomalies, and other diseases. As well; 28, 78, and 8% of them had inappropriate beliefs about duck meat, pineapples, and coconuts; respectively [38]. The results of the present study also showed a significantly direct relationship between adherence to nutritional behaviors and beliefs in the effectiveness of some food stuff on the neutralization of sugar intake in other ones, beliefs related to the consumption rate of some food stuff, beliefs associated with healthy foods, as well as spiritual beliefs. Besides, some diabetic patients believed that they could neutralize sugar released from foods high in starch through the consumption of herbal foods, barley flour, and sour stuff. Some of them similarly believed that low consumption of sugar, fats, and other unhealthy foods had no effects on their blood sugar levels and some other patients argued that sugar in some food stuff such as berries, raisins, dates, and honey or dairy fats had no negative impacts on health status as well as blood sugar levels. Consistent with the results of the present study, the findings of an investigation conducted in Pakistan highlighted a strong belief that diabetic patients were not allowed to have rooted plants because they were considered as sources of sugar and also believed that eating bread had no limitations provided that it had been made with pea flour [39]. A significantly inverse correlation was also observed between dietary adherence and exaggerated beliefs. Among the factors examined, the highest correlation was found between exaggerated beliefs and nutritional behaviors, so it could be concluded that the main factors affecting individuals to have unhealthy diets was exaggerated beliefs. In addition, the reviews of the related literature showed that wrong beliefs about “health value” of food stuff could also influence consumption patterns and trends. Accordingly, evaluating ready foods of high health value and having the belief that it could help in blood sugar control was accompanied by higher food intakes [40]. So, it became obvious that dietary beliefs could be found in all human communities wherein particular beliefs could be beneficial to all members or harmful for a special group [41]. As a result, awareness of beliefs within a society was assumed to determine the effectiveness of interventions to a great extent.

The patients’ sociocultural backgrounds could be also considered as a determinant of their success in managing diabetes [42]. Among such beliefs were spiritual ones which have been of importance since they can act as barriers to adherence to a diabetic diet [43]. Having extremist religious beliefs, some patients in this study believed that reliance on prophets as well as pilgrimage and visiting holy shrines even without practicing self-care activities could guarantee their health. In agreement with the results of this study, the findings of the investigation by Duke et al. [44] found that although spirituality could empower patients to cope with chronic diseases, it could possibly bring about concerns about diabetes care because these individuals assumed that their infliction with diabetes was related to their fate and that was also God’s act; thus, they avoided self-management as the result of this belief. In addition, the results of the study by Casarez et al. [45] showed that efforts to practice self-management activities including dietary adherence could be faded away. Another study on diabetic patients also revealed that strong beliefs in the control of the disease by God were accompanied by poor practice of self-care activities [46]. Thus, such beliefs could act against success if patients only focused on saying prayers or worshipping and ignored self-care activities [43]. However, such beliefs could sometimes raise hope and strengthen self-care. For example, the results of the study by Heidari et al. [47] showed a significantly positive correlation between scores of religious practices and those of self-care activities such as dietary adherence. Therefore, it could be claimed that spirituality and religion could be assumed as important factors affecting disease management in a way that extremes of excess and deficiency in terms of attention to these components in educational interventions to change self-care behaviors could lead to undesirable results.

Studies had further demonstrated that adherence to self-care behaviors by patients with T2D was really problematic and challenging since important points were required to be understood regarding this disease and there were even numerous fears and concerns in such patients [48]. Identifying the sources of such fears and concerns could thus contribute to the development of educational strategies. Following the educational intervention, the results of this study showed a significant fall in the mean scores of the sub-groups of fears, concerns, and discomforts associated with dietary adherence in the diabetic patients. Moreover, the results suggested a significantly inverse relationship between nutritional behaviors and concerns created by economic difficulties, fears, and concerns about the complications of adherence to a diabetic diet, concerns resulted from feeling like a burden and inattention by family members, and discomforts caused by life-changing miseries. Accordingly, concerns instigated by economic difficulties could be among the factors affecting dietary adherence in diabetic patients. This issue highlighted the fact that concerns arising from economic problems could affect patients’ decisions to purchase healthy food stuff and consequently adhere to a healthy diet. In line with the results of the present study, the findings of other investigations also showed that good economic status was positively correlated with dietary adherence and financial problems were inversely correlated with dietary recommendations [49]. Some patients also expressed their concerns that the diet advocated for diabetic patients was not complete enough and adherence to it could lead to various physical complications such as goiter, hair loss, upset stomach, anemia, and so on; thus, they were totally feeling anxious and terrified. The study by Munt et al. [50] similarly suggested that less self-regulation accompanied by a healthy diet was correlated with stronger beliefs towards discomforts caused by the consumption of healthy foods. In fact, most of the previous studies investigated fears associated with complications of the disease accompanied by non-adherence to diets [25, 51, 52] while the present study shed light on fears, concerns, and discomforts associated with adherence to a healthy diet considering the sociocultural backgrounds of the individuals within the society.

The results of this study also showed a significantly inverse relationship between adherence to a healthy diet and concerns about feeling like a burden and inattention by family members. It became obvious that diabetic patients were likely to be exposed to psychological tensions related to diabetes as they faced with the disease and its limitations considering their special conditions. So, in case of inattention by their family members, they might feel like a burden and go through more tensions and consequently pay less attention to self-care activities. Therefore, it was recommended to make use of educational interventions in the domain of family emotional support in order to moderate psychological tensions in such patients.

Furthermore; the results of this study indicated a reduction in the levels of FBS and HbA1c in the patients, 3 months after the educational intervention. Thus, the patients could achieve favorable theraputic objectives. In the investigation by Rusdiana et al. [53] on diabetic patients in Indonesia, a significant decrease was also found in the levels of blood sugar and HbA1c following an intervention. In addition, the results of a study conducted in the city of Kerman in Iran showed a falling trend in the levels of FBS and HbA1c, 3 months after the educational intervention in the intervention group [54] even though the levels of FBS in the control group significantly decreased in the present study. Almost certainly, the results of the primary tests in the control group acted as a warning and led to a dietary adherence by patients a few days before doing the second-stage tests which could reduce the levels of FBS because the average levels of HbA1c indicating the mean levels of patients’ blood sugar during 2 to 3 months [55] had also increased.

## Conclusion

The present study revealed significant results regarding the effectiveness of psychological factors related to adherence to a diabetic diet on improved performance of patients with T2D. Reduced levels of FBS and HbA1c in patients after the educational intervention compared to those before it also indicated the effectiveness of the educational intervention. Therefore, the psychological barriers identified in this study could serve as a basis for further research on factors affecting nutritional status, blood sugar control, as well as standardization of educational programs.

### Strengths and limitations of the study

(1) Among the strengths of the present study was the examination of fears, concerns, and discomforts associated with adherence to a diabetic diet and their introduction as barriers to such an adherence. It should be noted that previous studies reviewed had only shed light on the complications of diabetes due to lack of adherence to a diabetic diet as a facilitator. (2) In this study, a new combination of perceptions and beliefs of diabetic patients regarding healthy nutrition was investigated and their relationships with health outcomes were evaluated, which could be considered by healthcare workers and observers to treat patients. (3) One of the limitations of this study was that its findings could not be generalized to the entire Iranian diabetic population, because it was conducted in one geographical area. (4) In this study, it was not possible to simultaneously measure the effect of adherence to dietary behaviors and anti-diabetes drugs on the level of blood sugar in patients, so it was recommended to consider these issues in future studies.

### Suggestions for future research

Due to the fact that the Plate Method was a novel and easy way to boost self-efficacy in patients to adhere to a diabetic diet, it was recommended to separately use this strategy in future studies.

## Abbreviations

T2D: type 2 diabetes
FBS: fasting blood sugar
HbA1c: glycated hemoglobin
WHO: World Health Organization
PDAQ: Perceived Dietary Adherence Questionnaire
MAXQDA: qualitative data analysis software and Mac OS X
CVI: content validity index
CVR: content validity ratio
BMI: body mass index
